# Mesenchymal stem cells and porous β-tricalcium phosphate composites prepared through stem cell screen-enrich-combine(−biomaterials) circulating system for the repair of critical size bone defects in goat tibia

**DOI:** 10.1186/s13287-018-0906-1

**Published:** 2018-06-13

**Authors:** Wenxiang Chu, Yaokai Gan, Yifu Zhuang, Xin Wang, Jie Zhao, Tingting Tang, Kerong Dai

**Affiliations:** 1 Shanghai Key Laboratory of Orthopaedic Implants, Shanghai Ninth People’s Hospital, Shanghai Jiao Tong University School of Medicine, Shanghai, 200011 China; 2 Department of Orthopaedic Surgery, Shanghai Ninth People’s Hospital, Shanghai Jiao Tong University School of Medicine, Shanghai, 200011 China

**Keywords:** Critical size bone defects, Mesenchymal stem cells, Enrichment technique, Bone regeneration

## Abstract

**Background:**

Efficacious bone substitute is essential for the treatment of a critical size bone defect. Currently, the bone substitutes commonly used in clinical practice lack osteogenic capacity and the therapeutic efficacy is not ideal. Herein, a novel stem cell screen-enrich-combine(−biomaterials) circulating system (SECCS) was introduced to provide the substitutes with osteogenic ability. The stem cell screening, enrichment, and recombination with substitutes could be integrated during the surgical operation. Using SECCS, the bioactive mesenchymal stem cells (MSCs) and porous β-tricalcium phosphate (β-TCP) composites (MSCs/β-TCP) were rapidly prepared for critical size bone defect repair and validated in goat models of critical size tibia defects.

**Methods:**

Twelve goats with right hind limb tibia defects of 30 mm were randomly divided into two groups: six (the experimental group) were treated with MSCs/β-TCP prepared by SECCS and the other six goats (the control group) were treated with pure porous β-TCP. The repair effect was assessed by x-ray, computed tomography (CT), micro-CT, histology and histomorphology 6 months after the operation. In addition, the enrichment efficacy of MSCs and the characteristics of the MSCs/β-TCP prepared by SECCS were evaluated.

**Results:**

The SECCS could compound about 81.3 ± 3.0% of the MSCs in bone marrow to the porous β-TCP without affecting the cell viability. The average number of MSCs for retransplantation was 27,655.0 ± 5011.6 for each goat from the experimental group. In vitro, satisfactory biocompatibility of the MSCs/β-TCP was performed, with the MSCs spreading adequately, proliferating actively, and retaining the osteogenic potential. In vivo, the defect repair by MSCs/β-TCP with good medullary cavity recanalization and cortical remodeling was significantly superior to that of pure porous β-TCP.

**Conclusions:**

The MSCs/β-TCP prepared through SECCS demonstrated significant therapeutic efficacy in goat models of critical size bone defect. This provides a novel therapeutic strategy for critical size bone defects caused by severe injury, infection, and bone tumor resection with a high profile of safety, effectiveness, simplicity, and ease.

## Background

The current procedure for the treatment of critical size bone defects caused by severe injury, infection, bone tumor resection, and congenital deformity is not only challenging but also exhibits unsatisfactory outcomes [1–4]. Autogenous bone transplantation with effective repair outcome and a high safety profile has been regarded as the golden standard for bone defect repair; however, the limited resource of donor bones and the complications in donor sites such as bleeding and pain have restricted its clinical application [5–7]. Bone allografts and the processed products such as demineralized bone matrix have risks for immunological rejection and disease transmission, as well as lower osteogenic capability [8, 9]. Bioactive ceramic materials with osteoconduction properties such as porous β-tricalcium phosphate (β-TCP) and hydroxyapatite have been widely adopted in clinical practice, but the use of these materials is problematic for the repair of critical size bone defect [10–12].

Tissue engineering is another strategy for the treatment of critical size bone defects, and the effectiveness of bone engineering with mesenchymal stem cells (MSCs) as seeds for critical size bone defects has been demonstrated in several preclinical studies [3, 13–15]. Liu et al. [16] successfully repaired a 26-mm tibia defect in a goat model with in-vitro amplified MSCs and β-TCP composite (MSCs/β-TCP). Although satisfactory bone repair has been achieved using bone tissue engineering, the concerns regarding the ethics, safety, high cost, and long-term in-vitro culture have made its use in clinical practice challenging. On the contrary, stem cell enrichment techniques technically avoid the limitations of cell culture and effectively acquire bone marrow MSCs, which are the most critical components for osteogenesis. These could be used for bone repair by either a single injection or a combination with bone substitutes [17, 18]. Previous reports have demonstrated an efficacy of 95.1% for posterior spinal fusion with enriched bone marrow MSCs and β-TCP [19].

Conventional stem cell enrichment techniques are dependent on expensive cell sorters and are limited for clinical application. Based on the principles of high safety, effectiveness, minimal artificial interference, ease of use, and cost, a novel rapid enrichment system for bone marrow MSCs was developed by us utilizing the super adhesive capacity of stem cells. This could combine stem cell screening, enrichment, and recombination with substitute materials into one operational process. This novel system not only simplifies and standardizes the preparative process of MSC-based biomaterials, but also shortens the duration and reduces treatment cost.

**Fig. 1:**
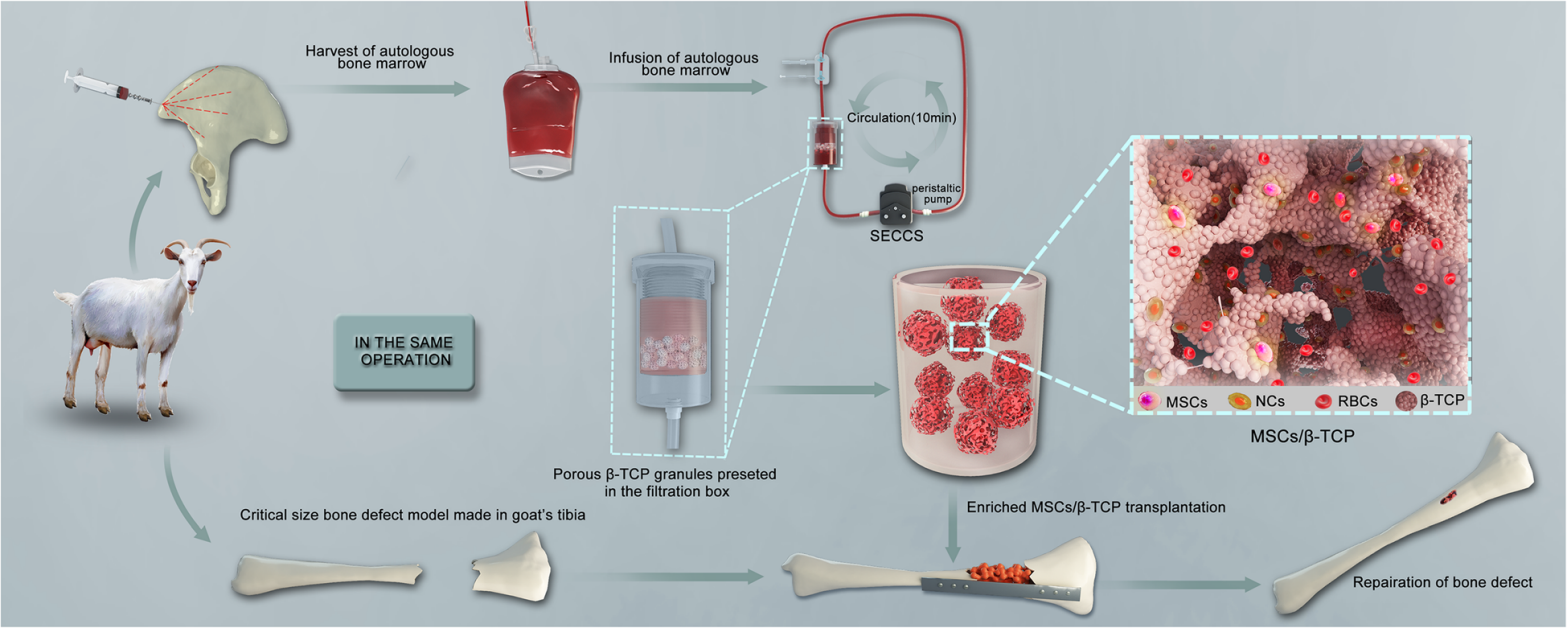
Stem cell screen-enrich-combine(−biomaterials) circulating system (SECCS) for preparing the mesenchymal stem cells and porous β-tricalcium phosphate composites (MSCs/β-TCP) for the treatment of critical size bone defects in the tibia of goats

In the current study, a stem cell screen-enrich-combine(−biomaterials) circulating system (SECCS) (patent number CN200710173407.4) is introduced as a bioactive material preparation system (Fig. 1). The repair effect of biomaterials prepared by this system was validated using a goat model with a critical size tibia defect. We demonstrate a new effective and simple option for the treatment of critical size bone defect.

## Methods

### Preparation of MSCs/β-TCP and animal surgery

All experimental procedures in this study were approved and performed in accordance with the guidelines of the Animal Ethics Committee of Shanghai Ninth People’s Hospital. Twelve goats with body weight ranging between 20 and 25 kg were used for this study. The experimental group (*n* = 6) was treated with MSCs/β-TCP, and the control group (*n* = 6) was treated with pure porous β-TCP. All goats were fasted for 24 h before surgery, and the surgery was performed under general anesthesia and electrocardiographic (ECG) monitoring. Bone marrow (50 ml) was obtained from each goat after anesthesia and collected in a blood bag containing 5000 U heparin, of which 3 ml was used for counting the number of colony-forming units that expressed alkaline phosphatase (CFUs/ALP^+^) and 1 ml was used for nucleated cell counting and cell viability measurements.

The SECCS consisted of one detachable columnar double-layer filter box, one control unit (controlling liquid infusion and aerofluxus by two three-way pipes), and one circulation loop containing a silastic tube for peristaltic pump working (Fig. 1). Five grams of porous β-TCP (Bio-Lu Bioceramics, Shanghai, China) was loaded into the inner filter box under sterile conditions and then the inner and outer filter box were successively screwed to the cap of filter box. After the whole circulation loop was connected and the three-way pipes were adjusted, about 45 ml of bone marrow was siphoned into the loop by inserting a trocar connecting with the infusion pipe into the blood bag. Then, the peristaltic pump was started and run at the frequency of 70–80 Hz to make the bone marrow stream slowly through the porous β-TCP. After 10 min of circulatory filtration, the three-way pipe was adjusted again for the processed marrow withdrawal. The MSCs/β-TCP composites within the inner box were taken out under sterile conditions. The bone marrow after filtration underwent the same tests as did the bone marrow before filtration.

After blood sampling, a 3-cm critical size bone defect was created between the middle and upper tibia. The periosteum from the defect region was then stripped. The defect was repaired using the prepared MSCs/β-TCP in the experimental group, and fixed in place by plate and screws before wound suture and dressing. Similar treatment procedure was performed for the control group except the defect was repaired using pure porous β-TCP.

### CFU/ALP^+^, nucleated cell counts, and cell viability in bone marrow before and after enrichment

The bone marrow samples prior to and after enrichment were seeded into six-well plates at a density of 0.1 ml/well, respectively; 2 ml of osteogenic inducer (α-minimum essential medium (αMEM) supplemented with 10% fetal bovine serum (FBS), 0.1 mM dexamethasone, 50 mM ascorbic acid, and 10 mM β-glycerophosphate sodium (Sigma)) was then added and the cells were cultured in the incubator with 5% CO_2_ at 37 °C. The culture medium was changed with fresh medium every 2 days. Alkaline phosphatase (ALP) staining was performed at day 14 after seeding and the number of CFUs/ALP^+^ were counted. The average number of CFUs/ ALP^+^ colonies in the three wells multiplied by 10 was taken as the number of MSCs in 1 ml of bone marrow blood.

One milliliter of bone marrow was added to 8 ml of erythrocyte lysate, mixed well, and incubated for 5 min before centrifugation for 5 min at 300 g. The supernatant was discarded, and the cells were resuspended in 10 ml serum-free medium. The process was repeated two to three times. The cells were then resuspended in 1 ml serum-free medium, and 10 μl of the cell suspension was mixed with 10 μl Trypan blue dye for nucleated cell counting and cell viability detection using the Countess® II (Thermofisher) instrument.

### MSC culture, and osteogenic, adipogenic and chondrogenic induction and identification

Two milliliters of bone marrow was mixed with 10 ml complete culture medium (α-MEM (Hyclone) containing 10% (v/v) FBS (GIBCO) and antibiotics (penicillin 100 U/mL, streptomycin 100 mg/mL)) and seeded into 10-cm Petri dishes and incubated at 37 °C with 5% CO_2_. The culture medium was changed every 3 days and passaged for 12 days until colonies were large enough.

First-generation MSCs were seeded into six-well plate and the culture medium was changed to osteogenesis induction medium when the cells reached 80% confluency. The culture medium was then changed every 2 days until day 21 when Alizarin red staining was performed after osteogenesis induction. The cultures were washed once with phosphate-buffered saline (PBS) and fixed with 4% paraformaldehyde for 20 min. Then the cells were washed with PBS twice and Alizarin red staining was performed at 37 °C for 30 min.

Adipogenic induction was performed as previously described [20]. In brief, confluent cells were cultured with adipogenic induction solution A (HUXMA-90031, Cyagen) for 3 days and switched to adipogenic induction solution B (HUXMA-90031, Cyagen) for 1 day, and then cultured for 21 days before Oil red staining. The culture medium was discarded and the cells were washed with PBS once, followed with 4% paraformaldehyde fixation for 20 min. The cells were washed with PBS before incubating the cells with Oil red solution for 30 min at 37 °C.

Aliquots (0.5 ml) of MSCs at a density of 5 × 10^5^/mL were transferred to 15-ml tubes and centrifuged for 5 min at 300 g. Then, 0.5 ml chondrogenic induction solution (HUXMA-90041, Cyagen) was added into the tube and incubated for 48 h at 37 °C with 5% CO_2_. The cell pellets were flipped into the culture medium after 48 h and the medium was changed every 3 days. At day 28, the cells were fixed with 4% paraformaldehyde followed with dehydration for tissue slice preparation before Alcian blue staining.

### In-vitro amplification and osteogenic differentiation of MSCs from MSCs/β-TCP

Fifteen particles of well-prepared MSCs/β-TCP were rinsed with PBS and seeded into 12-well plates. Particles were then incubated with 1 ml complete culture medium at 37 °C with 5% CO_2_, with medium change every 2 days. CCK8 detection reagents (100 μl) were added into the culture medium 3 h before a medium change, and then distributed into 96-well plates after incubation. Each well contained 100 μl and the final readout was performed using eight replicate wells by measuring the absorption at 450 nm. The MSCs/β-TCP were washed with PBS thoroughly and replaced with 1 ml complete culture medium. The same procedure was performed for the samples from the pure porous β-TCP.

The complete culture medium of the MSCs/β-TCP was changed to osteogenic induction medium 1 week later, and then cultured for an additional 2 weeks. The cells were then washed with PBS twice and fixed with 4% paraformaldehyde for 20 min. The MSCs/β-TCP were stained with ALP staining reagent and incubated at 37 °C for 1 h.

### MSCs/β-TCP observation

The freshly prepared MSCs/β-TCP and MSCs/β-TCP after 2 weeks of in-vitro culture were gently washed with PBS, followed with glutaraldehyde fixation overnight at 4 °C and observed under a scanning electron microscope (SEM) after gradual dehydration and metal spraying. The MSCs/β-TCP after 2 weeks of culture in complete medium were fixed with 4% paraformaldehyde, followed with three washes with PBS. The cultures were then incubated with 0.5%Triton-100 for 5 min and then washed with PBS for 30 s. Cells were incubated with rhodamine at room temperature for 1 h in the dark. After incubation, the cells were washed three times in PBS and then incubated with DAPI stain for 10 min. Afterwards, the cells were washed and observed under a confocal microscopy.

### Critical size bone defect repair evaluated by x-ray, computed tomography (CT), micro-CT, histology, and histomorphology

All animals were evaluated by x-ray and CT scans 1 week and 6 months after surgery. The right hind limb tibia of each animal was then removed and scanned using micro-CT followed by three-dimensional (3D) image reconstruction.

Bone samples were embedded in polymethyl methacrylate and then sliced to 100 μm thickness for slide preparation and Van Gieson’s picric–fuchsine staining. Three samples with three sections in each sample were randomly selected from both groups for morphometrical analysis, and new bone regions as well as residual material regions were calculated using Image-Pro Plus 6.0.

### Statistical analysis

All data were processed using the SPSS24.0 software. Matched pair data were compared using *t* tests, and group comparison was analyzed using group *t* tests. All data are presented as mean ± SD. *p* < 0.05 was considered as statistically significant.

## Results

### Effect of enrichment on bone marrow MSCs using SECCS

**Fig. 2:**
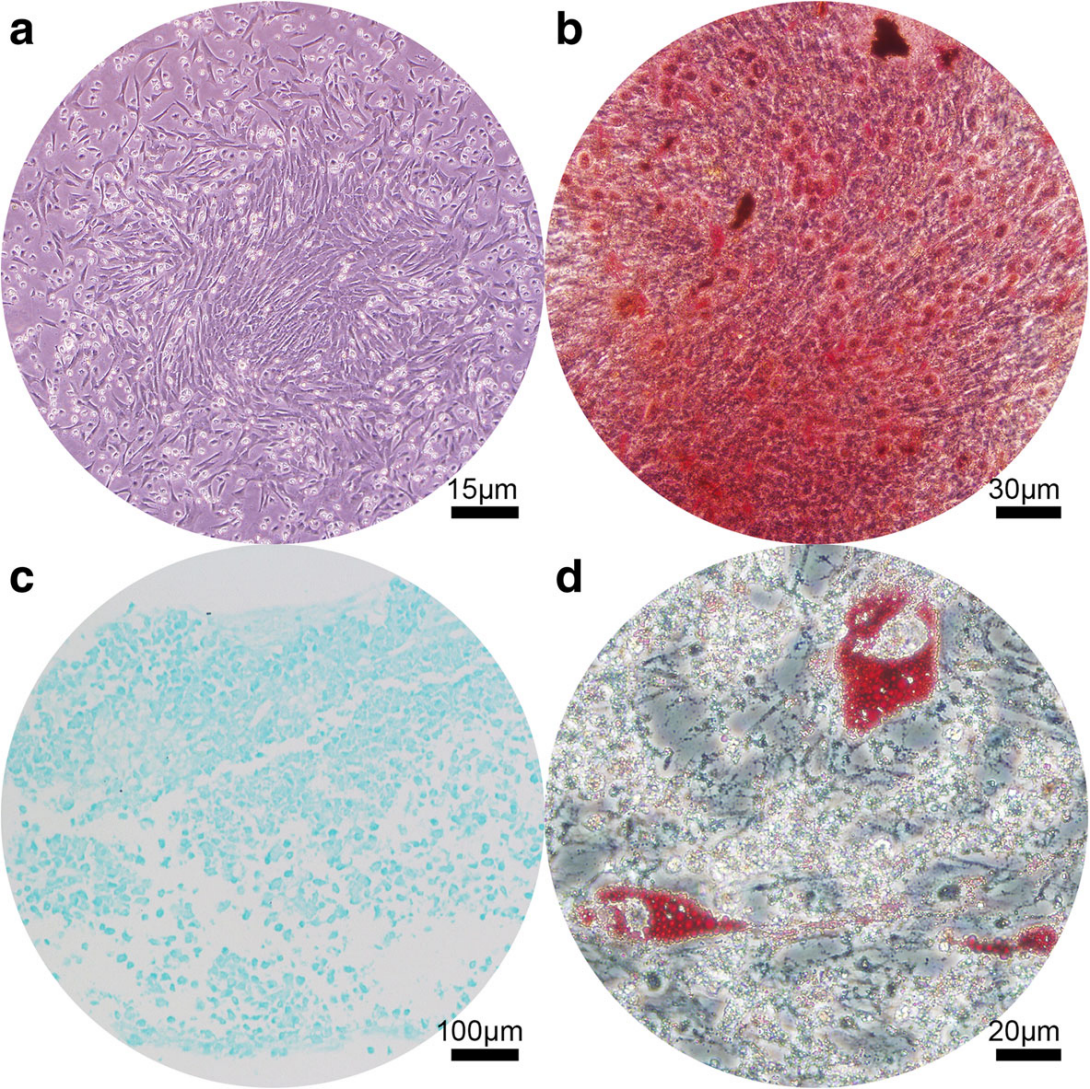
Evaluation of multilineage differentiation ability of goat bone marrow mesenchymal stem cells. **a** Adherent long spindle-shaped cell colonies after 7 days of culture; **b** Alizarin red staining after 21 days of osteogenic differentiation induction; **c** Alcian blue staining after 28 days of chondrogenic induction; **d** Oil red staining after 21 days of adipogenic induction

Long spindle-shaped cell colonies were formed from bone marrows after 7 days of culture (Fig. 2a). The adherent cells were trypsinized and passaged, and the osteogenic (Fig. 2b), chondrogenic (Fig. 2c), and adipogenic (Fig. 2d) differentiation ability was observed after induction. The ability for three-line differentiation confirmed the MSC characteristics.

**Fig. 3:**
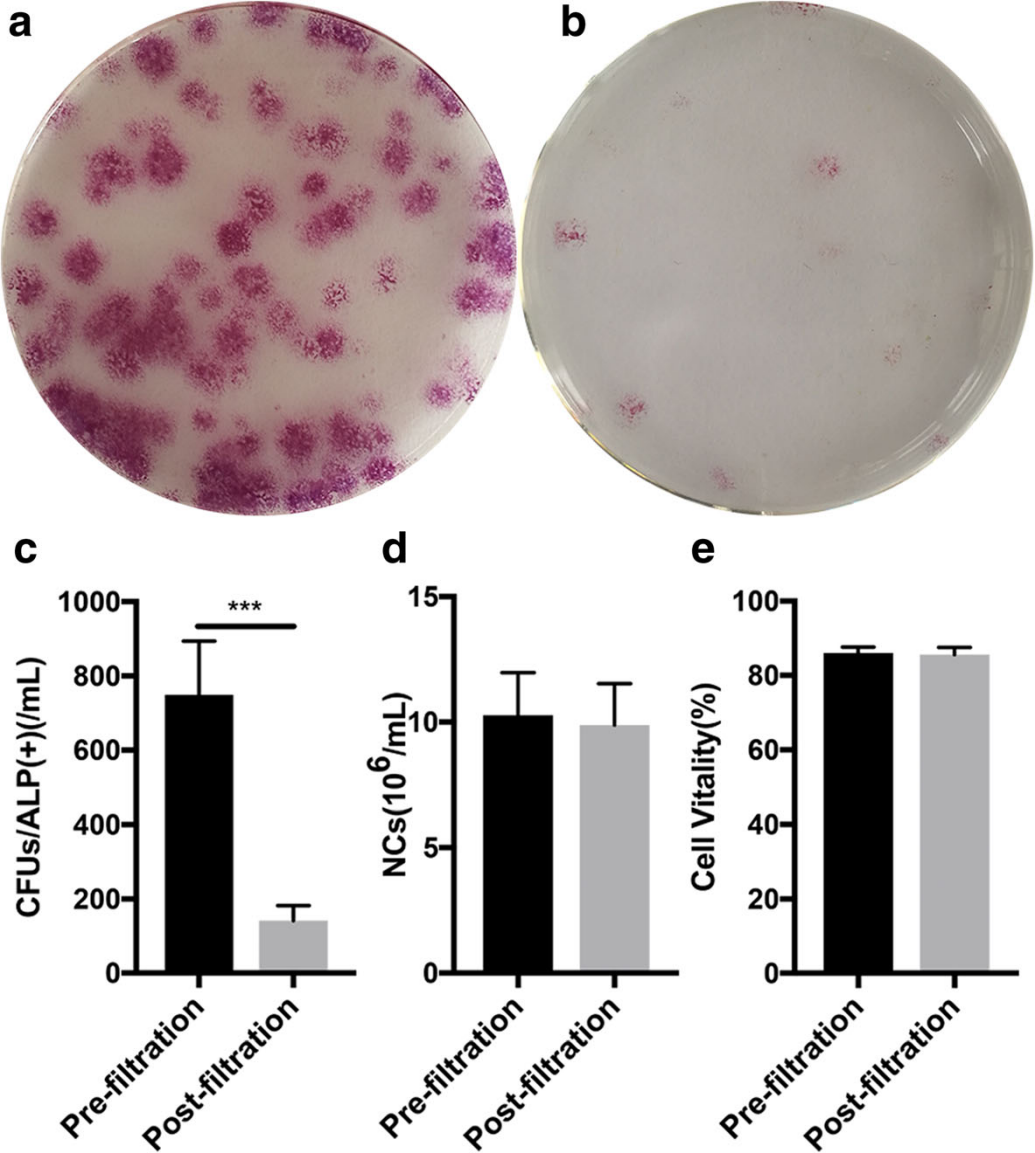
Enrichment efficiency of SECCS and its effect on cell viability. **a,b** ALP staining to evaluate MSCs (CFU/ALP^+^) before and after bone marrow filtration. **c,d** quantitative analysis of MSCs (**c**) and nucleated cells (**d**) before and after bone marrow filtration; **e** Trypan blue staining to evaluate cell viability before and after filtration

The number of CFU-ALP^+^ in the bone marrow was 749.2 ± 144.8/ml and 141.2 ± 41.3/ml before and after filtration, respectively. The number of CFU-ALP^+^ was significantly reduced after filtration (*t* = 13.4, *p* < 0.01; Fig. 3a–c) and the adherent rate was 81.3 ± 3.0%. The average number of MSCs for retransplantation was 27,655.0 ± 5011.6 for each goat from the experimental group. However, the number of nucleated cells in the bone marrow did not reduce significantly from 10.3 ± 1.7 × 10^6^/ml before filtration to 9.9 ± 1.6 × 10^6^/ml after filtration (*p* = 0.074; Fig. 3d). The filtration process had no significant effect on cell viability (86.1 ± 1.6% versus 85.6 ± 2.0%; *t* = 1.191, *p* > 0.05; Fig. 3e).

### Structural features and in-vitro bioactivity of MSCs/β-TCP

**Fig. 4:**
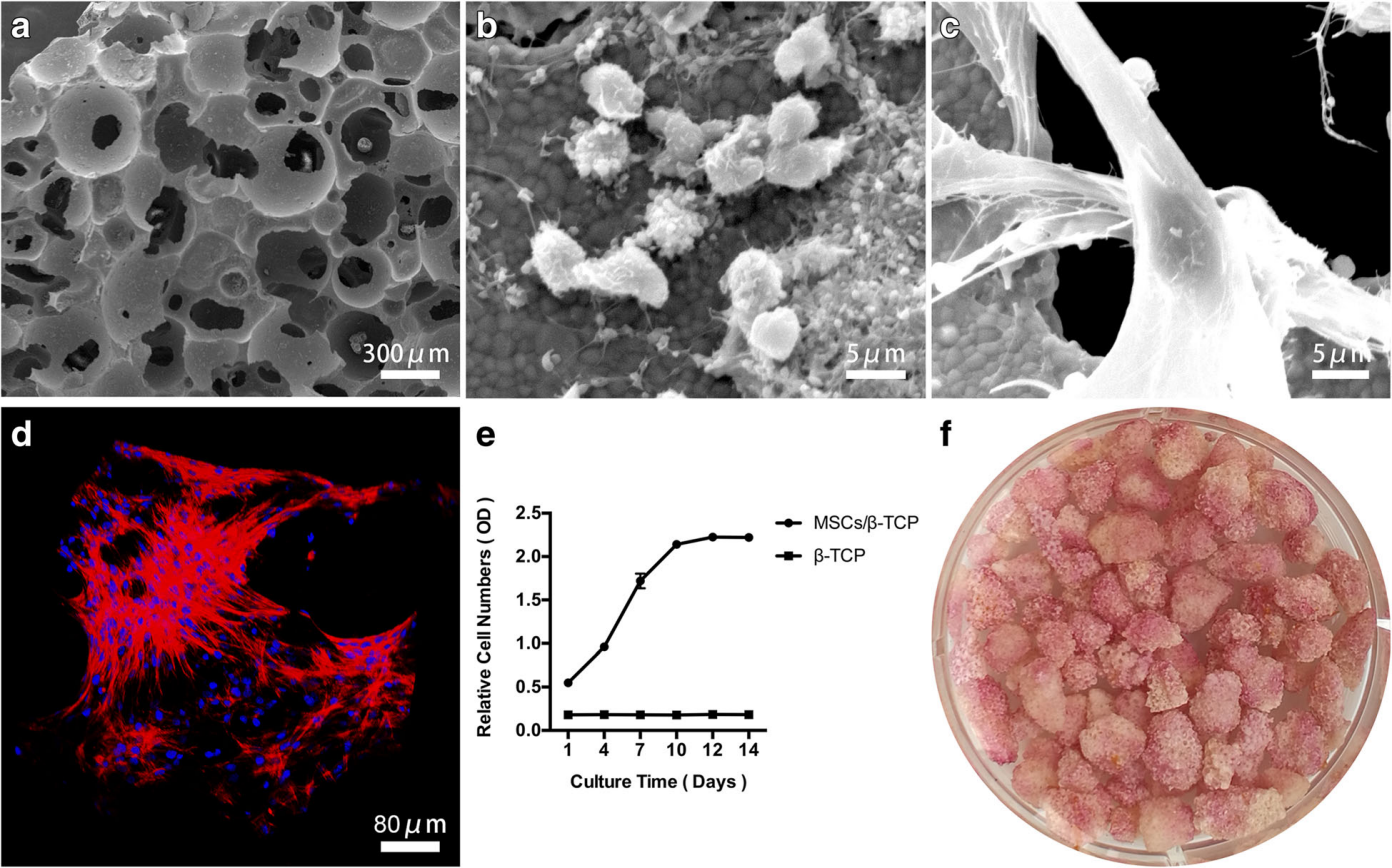
Adherence, proliferation, and osteogenic differentiation of MSCs from mesenchymal stem cells and porous β-tricalcium phosphate composites (MSCS/β-TCP). **a** Porous β-TCP. **b,c** Electron micrograph of MSCs/β-TCP cultured for 6 h and 10 days. **d** Large number of cells spread on the inner wall and surface of β-TCP from MSCs/β-TCP after 10 days of culture. **e** Cell proliferation in MSCs/β-TCP evaluated by CCK8. **f** ALP staining for MSCs/β-TCP after 14 days of osteogenic induction. OD optical density

β-TCP particles were observed as porous structures under SEM, with pores distributed randomly and connected with each other in various sizes and shapes (Fig. 4a). After rapid screening and enrichment, MSCs could adhere to the inner walls of the pores (Fig. 4b). The adherent cells of MSCs/β-TCP were spread as long spindles within the inner and outer surfaces of the particles after 2 weeks of culture (Fig. 4c). The MSCs were widely distributed around the internal wall of particles under confocal microscopy, and the cells connected with each other (Fig. 4d).

The adherent cells within the porous β-TCP particles could rapidly proliferate under the in-vitro culture conditions and stability could be achieved after 12 days of culture with an ‘S’ shaped amplification curve (Fig. 4e). The large amount of ALP produced by MSCs/β-TCP after 14 days of osteogenic induction (Fig. 4f) indicated that the MSCs adhering to the porous β-TCP particles still retained osteogenic potential.

### Repair efficacy of critical size bone defect in the goat model

**Fig. 5:**
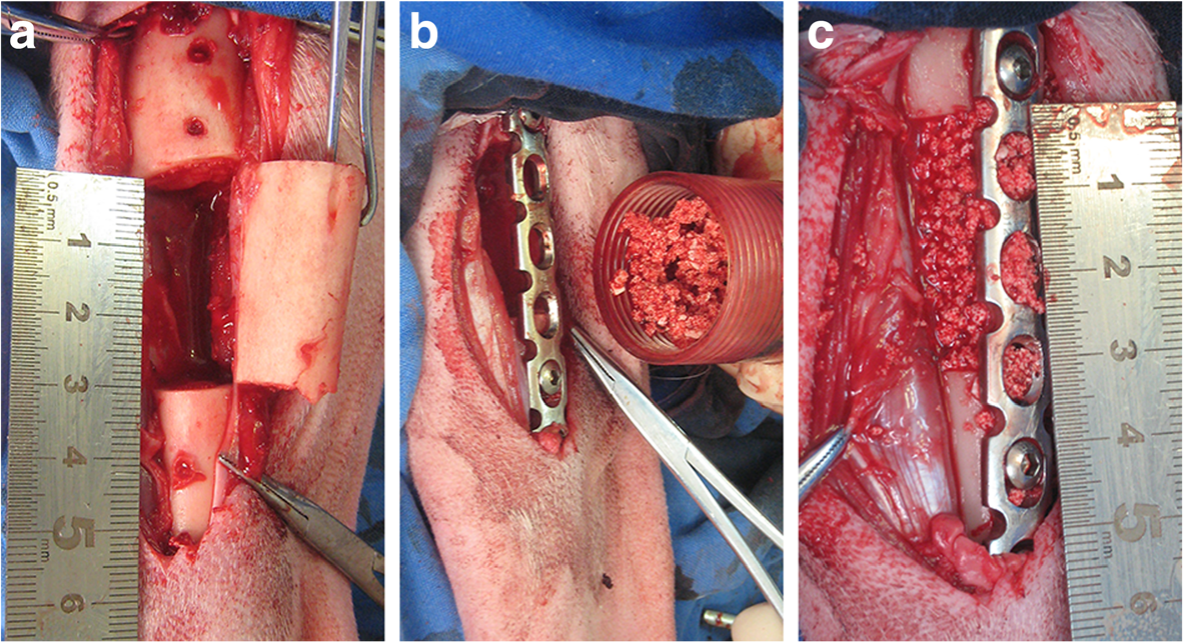
**a** Critical size bone defect (3 cm) in tibia of goat. **b,c** MSCs/β-TCP substitute treatment

**Fig. 6:**
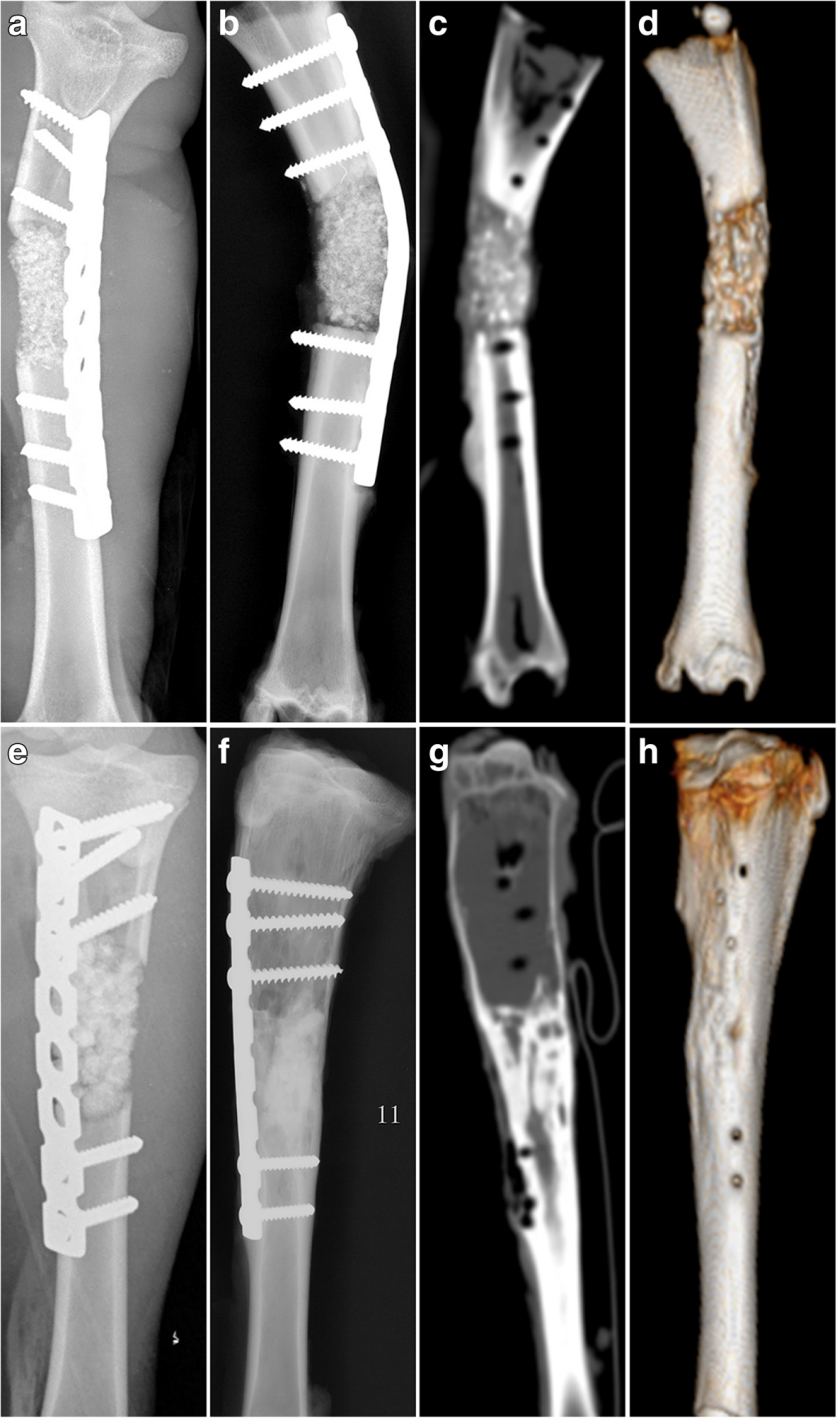
Images of critical size bone defect in goat tibia. X-ray films of pure β-TCP substitute treatment at **a** 1 week and **b** 6 months after the operation. **c** CT scans of pure β-TCP substitute treatment at 6 months with **d** 3D reconstruction image. X-ray films of MSCs/β-TCP substitute treatment at **e** 1 week and **f** 6 months after the operation. **g** CT scans of MSCs/β-TCP substitute treatment at 6 months with **h** 3D reconstruction image

The goat model for critical size bone defects was established by the removal of a 3-cm middle and upper tibia fragment (Fig. 5a). The defects were filled with either MSCs/β-TCP (Fig. 5b, c) or pure β-TCP, and fixed in place with metal plates and screws. There was one goat in the experimental group that developed incision infection and was not included in the study; the remaining goats recovered well 6 months after the operation. Callus formation, cortical continuity, and blurred images of TCP were observed by x-ray and CT scan (Fig. 6f–h), whereas condensed particle-like images of TCP were observed in the control group (Fig. 6b–d) with obvious boundaries between the broken end and the normal bone tissue, which indicated that the bone defect area was not repaired.

**Fig. 7:**
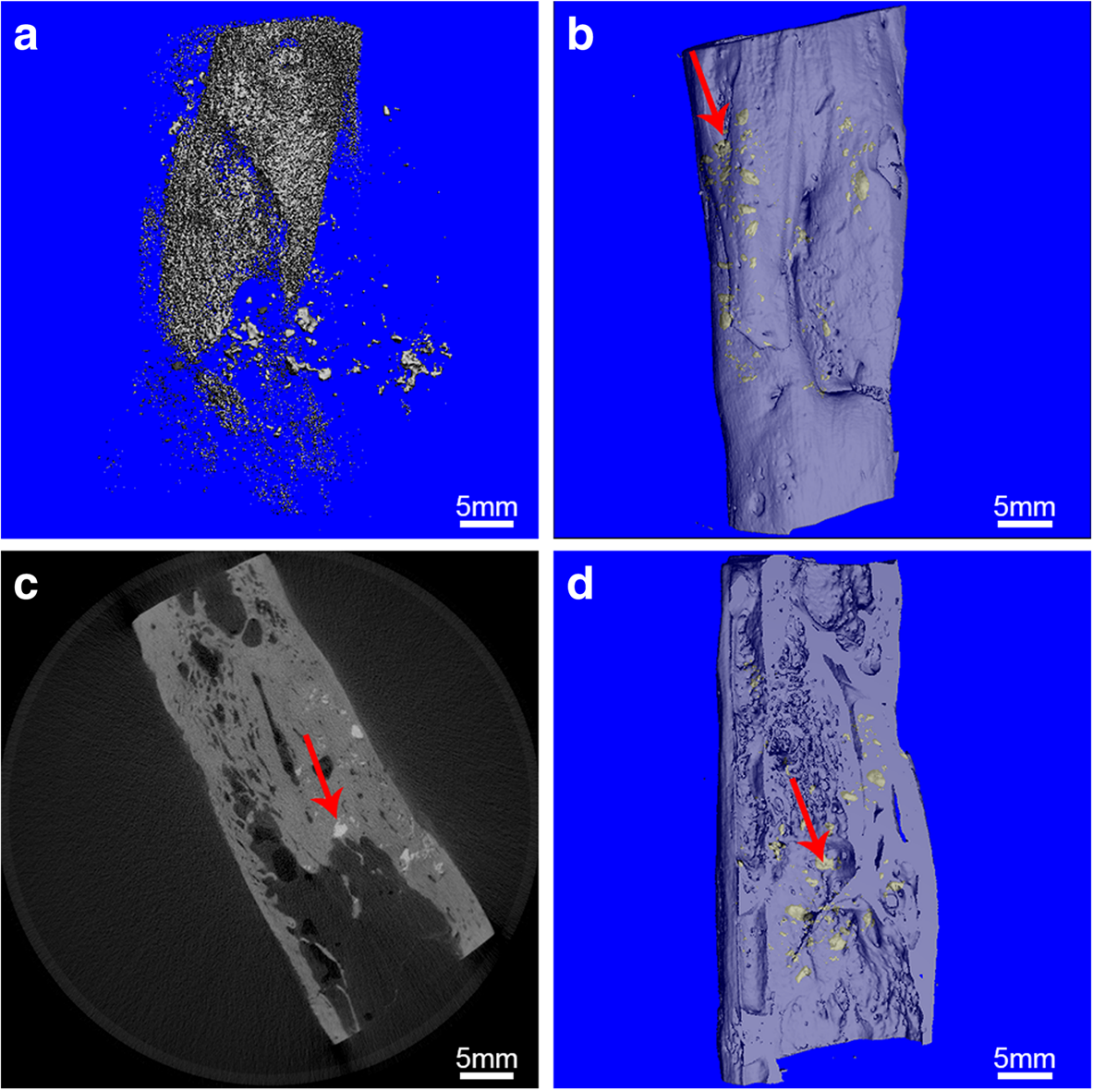
Micro-CT scans of bone defects. **a** Image of pure porous β-TCP substitute at 6 months postoperation with 3D reconstruction; **b–d** image of MSCs/β-TCP substitute at 6 months postoperation with 3D reconstruction. The red colored arrow indicates residual β-TCP

It was also revealed by micro-CT scan that the TCP was still present as particles after 6 months in the control group with an absence of osteogenesis (Fig. 7a); however, lamellar bone tissues were observed after MSCs/β-TCP transplantation in the experimental group, with only a few nondegraded TCP. The longitudinal section demonstrated that the medullary cavity was reconnected and the bone defect was completely repaired by the regenerated bone tissue with healthy integration of normal bone tissue at both ends (Fig. 7b–d).

**Fig. 8:**
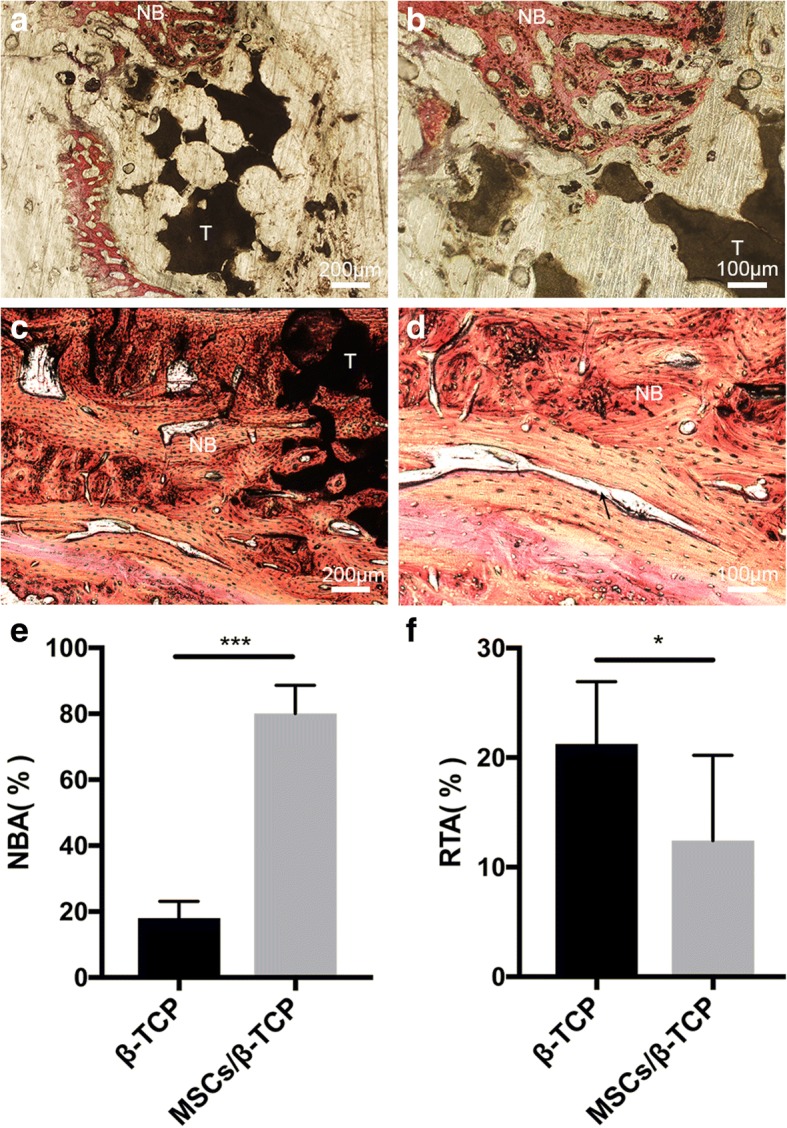
Histology and histomorphology evaluation. Van Gieson staining of hard tissue slices 6 months after operation from pure porous β-tricalcium phosphate (TCP) treatment (**a,b**) and mesenchymal stem cells and porous β-tricalcium phosphate composites (MSCs/β-TCP) (**c,d**) treatment of 3-cm bone defects. Black arrow indicates new Haver’s tube formation. **e,f** Percentage of new bone area (NBA) and residual β-TCP area (RTA). NB new bone tissues, T residual β-TCP. *p < 0.05, ***p < 0.01

Hard tissue slices of the repaired regions were used for evaluating new bone regeneration after pure β-TCP or MSCs/β-TCP implantation. The new bone tissues were restricted to the margins of the pure β-TCP implantation site and a large amount of nondegraded β-TCP particles remained in the middle of the implantation area (Fig. 8a, b), whereas new bone tissues regenerated in the MSCs/β-TCP transplantation site which was compact and firm (Fig. 8c, d). In addition, there was a lamellar bone formation in the experimental group, with well-reconstructed luminal structures of the Havers’ system (Fig. 8d).

Quantitative measurements indicated that the area of new bone formation was significantly larger in the experimental group compared with the control group (Fig. 8e) (80.1 ± 8.6% vs 18.0 ± 5.1%; *t* = 18.632, *p* < 0.01), and the residual implantation material was significantly lower in the experimental group compared with the control group (12.4 ± 7.8% vs 21.3 ± 5.7%, *t* = 2.750, *p* = 0.014) (Fig. 8f).

## Discussion

Critical size bone defects are too large to be repaired naturally and depend on substitute material implantation for proper correction of the defect [21, 22]. It has been demonstrated in preclinical studies that bone marrow MSCs could aid in the repair of bone defects [23–25]. Tissue engineered bones constructed by seed cells obtained from in-vitro amplified bone marrow MSCs have demonstrated repair efficacy in multiple animal models with critical size bone defects. However, the risk of chromosomal mutations and microorganism contamination has remained a serious concern for in-vitro cell culture and amplification of MSCs, and hence has not been fully accepted for clinical application. In the current study, we developed SECCS, which we used to enrich MSCs from bone marrows during surgery with the MSCs being rapidly integrated with porous β-TCP to repair critical size tibia defects in goat models. The maturation of tubular bone tissues was observed in our study and our findings provide a new strategy for critical size bone defect repair.

MSCs have the potential for multilineage differentiation (osteogenic, adipogenic and chondrogenic differentiation), and act as seed cells with broad applications [26–29]. MSCs account for only 0.001–0.01% of total bone marrow nucleated cells [30] and can be separated from the other cell types by taking advantage of their propensity to adhere to plastic surfaces. In the development of the stem cell enrichment technique, bone marrow MSCs were found to directly adhere to the surface of the filter strainer, which was originally used to separate blood clots in the bone marrow when bone marrow passed through it. This inspired us to develop the stem cell screen-enrich-combine(−biomaterials) circulating system (SECCS) for bone marrow MSC enrichment. In the SECCS system, bone marrow is filtered through a porous substitute material to which MSCs within it could adhere, hence accomplishing the one-step process of screening, enrichment, and combination with substitute material in the preparation of the bioactive material. In the current study, bone marrows were processed using SECCS and the remaining cells were analyzed using CFU-ALP^+^ counting. It was found that MSCs were significantly recovered and enriched by SECCS with efficiencies of 81.3 ± 3.0% (Fig. 3a–c). However, most of the nucleated cells directly passed through the porous material together with the bone marrow liquid (Fig. 3d), which indicates the exclusive adhesive property of MSCs. In addition, SEM further demonstrated that MSCs successfully adhered to the inner wall of the substitute material (Fig. 4b), indicating that SECCS could rapidly screen, enrich, and integrate MSCs with the substitute material in one process. The number of MSCs that are used affect bone formation [31–33]. In the current study, 27,655.0 ± 5011.6 MSCs were successfully enriched from each goat. Although much lower than cell numbers obtained from in-vitro culture, this number was sufficient to successfully repair 3-cm bone defects. Using conventional in vitro cell culture technology, the cell density from in-vitro culture could not match the initial seeding density because of the sudden drop in nutrient supply or the sudden change in the cell survival environment [32]. It took only 10 min for the filtration process to enrich MSCs using SECCS without any exogenous reagents involved, and hence minimized the manipulation of MSCs [34]. Using the SECCS method, cell viability and bioactivity could be preserved. This was confirmed by comparing the cells before and after filtration (Fig. 3e). The high viability and relatively sufficient nutrient supply enabled MSCs to proliferate rapidly in vivo.

Good biocompatibility is a key factor for bioactive materials to exert an efficacious outcome. Porous β-TCP has good biodegradability and is commonly used as a substitute for bone grafts [35, 36]. In our study, the unique porous structure of β-TCP enables bone marrow cells to integrate into them effectively (Fig. 4a) and provides sufficient space for MSCs to adhere, hence making for a suitable cell carrier. MSCs/β-TCP prepared using SECCS were cultured in vitro for 10 days and adhered to the inner walls of the β-TCP as long spindle-like cells (Fig. 4c). This was confirmed by confocal microscopy demonstrating a large number of MSCs spreading around the inner walls of the substitute material, with connections among the cells (Fig. 4d). The good spreading capability of stem cells was a vital prerequisite for osteogenic differentiation. Limited contractility could significantly inhibit proliferation and osteogenic differentiation of MSCs [37]. In our study, β-TCP pores were 500 µm in diameter with 70% porosity, which provided an ideal 3D space for cell proliferation and spreading. In addition, MSCs/β-TCP had good proliferation capacity during in-vitro culture, with an “S” shaped proliferation curve (Fig. 4e) indicating that cell proliferation could be rapidly initiated under the conditions of sufficient nutrient supply. Osteogenic capability is another key factor of the bioactive material. MSCs/β-TCP produced large amounts of alkaline phosphatase after osteogenic induction (Fig. 4f), suggesting that the MSCs enriched using SECCS had good compatibility and good osteogenic ability.

Critical size bone defect models were established by Liu et al. on goats which were 22.3 ± 4.1 kg in weight and had 26-mm bone defects [16]. In our experiment, a 30-mm bone defect was established in the tibia of goats of similar body weight compared with the study of Liu et al. In our study, the common limit of the bone defect was far larger. MSCs/β-TCP prepared using SECCS demonstrated satisfactory bone repair efficacy. Six months after MSCs/β-TCP, the substitute materials observed by x-ray were blurred indicating absorption of the substitute material. In addition, CT scans revealed continuous cortical bone formation and recanalization of the medullary cavity in the new bone areas (Fig. 6g–h). More detailed images revealed by micro-CT demonstrated that the new bone areas had formed structures resembling normal tubular bone, including the peripheral cortical bone, spongy bone, and penetrating medullary cavity (Fig. 7b–d). Both continuous cortical bone and reconnecting medullary cavity are important characteristics of normal tubular bone structure.

Mechanical stress plays an important role in bone structure remodeling [38–40]. The remodeled cortical bone was closely connected to the adjacent normal bone, suggesting that the internal fixation plates played a significant role in stress conduction and facilitated the remodeling of the regenerated bone. It was demonstrated by Petite et al. [32] that in-vitro cultured MSCs inoculated onto the coral scaffold and filled into critical 25-mm size defects in goat metatarsal models followed with plate fixation had good medullary cavity recanalization and cortical remodeling 16 weeks after surgery. In our study, the enriched 27,655.0 ± 5011.6 MSCs reinforced the osteogenic capability of the β-TCP, and the MSCs/β-TCP significantly enhanced new bone regeneration. MSCs/β-TCP were observed to degrade as reflected by histology and histomorphology (Fig. 8c–f). In addition, there was lamellar bone formation in the experimental group, including well-reconstructed luminal structures of the Havers’ system (Fig. 8d) suggesting good bone regeneration induced by MSCs/β-TCP.

However, obvious particles were observed after 6 months in the control group treated with pure porous β-TCP, with poor bone healing observed by CT scans (Fig. 6c–d). The histological staining of tissue slices showed that there were large amounts of residual substitute materials, with only a few new bone formations at the margin areas (Fig. 8a, b). It was reported that porous β-TCP could be utilized alone as a bone substitute material for the treatment of bone defects and was degradable when new bone was formed; however, it does not have osteogenic capability, and the bone defect repair had to depend on the creeping substitution of surrounding new bone tissues with limited repair ability [41–43]. Our findings showed that in the control group there was 21.3 ± 5.7% residual β-TCP and only 18.0 ± 5.1% new born formation, implying that most of the porous β-TCP in bone defect areas was absorbed and not able to form new bone tissue with enough bone mass. Hence, pure porous β-TCP cannot be used alone for 3-cm bone defect treatment in goat tibias, whereas MSCs/β-TCP had the powerful bone regeneration capability with 80.1 ± 8.6% new bone formation in 6 months.

The safety concern is another important factor during implantation and cellular therapy. SECCS is a closed loop design and effectively insulates against microbial contamination. This is important to maintain sterility during surgery. There was only one goat that died of incision infection after MSCs/β-TCP treatment, which may be due to wound licking. Compared with conventional in-vitro cell culture, SECCS effectively avoids exogenous reagents [44] and the ethical issue regarding the use of animal serum, as well as the potential safety risk related to long-term in-vitro culture [45, 46]. In addition, SECCS could rapidly enrich bone marrow MSCs and integrate them into porous bioactive materials. This could be used during surgery since it only takes about 10 min to integrate. Thus, this procedure greatly benefits urgent surgical bone repair.

SECCS is a bioactive material preparation system with a high safety profile, effectiveness, and convenience, and could be used during the surgical procedure to enrich autologous bone marrow MSCs and integrate them with conventional bone substitutes for osteoinduction and osteogenesis. Given the satisfied efficacy achieved using SECCS for goat models with critical size bone defects, the therapeutic efficacy in humans is currently being investigated in randomized controlled clinical trials (clinical trial registration no. ChiCRT-INR-16009871).

## Conclusion

SECCS integrates the three main steps of MSC screening, enrichment, and combination with substitute materials and could be used during a surgical procedure. This method could incorporate MSCs with conventional bone substitutes rapidly to enable them to have good osteogenic ability. MSCs/β-TCP composite could effectively repair critical size defects in the tibias of goats. As a novel bioactive material preparation strategy, SECCS could provide a new procedure to repair critical size bone defects. SECCS has a high safety profile, effectiveness, rapidness, low cost, and easy application.

## Abbreviations

ALP: Alkaline phosphatase
CT: Computed tomography
CFUs/ALP^+^: Colony-forming units that expressed alkaline phosphatase
ECG: Electrocardiographic
FBS: Fetal bovine serum
MEM: Minimum essential medium
MSC: Mesenchymal stem cell
MSCs/β-TCP: Mesenchymal stem cells and porous β-tricalcium phosphate composites
PBS: Phosphate-buffered saline
SECCS: Stem cell screen-enrich-combine(−biomaterials) circulating system
SEM: Scanning electron microscope
TCP: Tricalcium phosphate
